# Functionalized gold nanorod nanocomposite system to modulate differentiation of human mesenchymal stem cells into neural-like progenitors

**DOI:** 10.1038/s41598-017-16800-9

**Published:** 2017-11-30

**Authors:** Karrer M. Alghazali, Steven D. Newby, Zeid A. Nima, Rabab N. Hamzah, Fumiya Watanabe, Shawn E. Bourdo, Thomas J. Masi, Stacy M. Stephenson, David E. Anderson, Madhu s. Dhar, Alexandru S. Biris

**Affiliations:** 1 Center of Integrative Nanotechnology Sciences, University of Arkansas at Little Rock, Little Rock, AR 72204 USA; 2 College of Veterinary Medicine, University of Tennessee, Knoxville, TN 37996 USA; 3 University of Tennessee Graduate School of Medicine, Knoxville, TN 37996 USA

## Abstract

A 2D multifunctional nanocomposite system of gold nanorods (AuNRs) was developed. Gold nanorods were functionalized via polyethylene glycol with a terminal amine, and, were characterized using transmission and scanning electron microscopy, ultra violet-visible and X-ray photoelectron spectroscopy, and Zeta-potential. The system was cytocompatible to and maintained the integrity of Schwann cells. The neurogenic potential of adipose tissue – derived human mesenchymal stem cells (hMSCs) was evaluated *in vitro*. The expression pattern and localization of Vimentin confirmed the mesenchymal origin of cells and tracked morphological changes during differentiation. The expression patterns of S100β and glial fibrillary acidic protein (GFAP), were used as indicator for neural differentiation. Results suggested that this process was enhanced when the cells were seeded on the AuNRs compared to the tissue-culture surface. The present study indicates that the design and the surface properties of the AuNRs enhances neural differentiation of hMSCs and hence, would be beneficial for neural tissue engineering scaffolds.

## Introduction

Bionanotechnology presents a revolutionary new approach to regenerative medicine and tissue engineering. Nanotechnology offers the possibility of creating tunable surface chemistry and adjustable size and surface area on a biocompatible substrate—features that can be extremely useful for complex biomedical applications. The interaction between a biocompatible substrate and a cell membrane has been cited as an essential control factor in cellular fate^1^. The therapeutic action of regenerative devices is a function of their chemistry profile, which factors into their control and enhancement of the tissue regeneration process^2,3^. In turn, this essential chemistry profile facilitates the selection process of loading the necessary components within the biocompatible substrate^4^. Therefore, the behavior of nanomaterials should be thoroughly understood when considering them for use as therapeutic aides for biological responses.

Previously, significant work has been conducted to explore the use of nanoparticles (NPs) in regenerative medicine, especially in the application of the NPs’ electrical, chemical, and other unique properties for neural tissue regeneration. For example, Ciofani and his team have established electrical stimulation treatment of cells that relies on the piezoelectric behavior of boron nitride nanotubes. Using this method, they saw a 30% increase in axonal outgrowth of the PC12 cells absorbed after 9 days of treatment compared to the control^5^. Gold NPs have also been used to modify the surface of a bioactive substrate—a chitosan-gold NP system was grafted onto poly(D,L-lactide) to design nerve conduits, and the resulting system improved the regeneration process of an *in vivo* transection model of the rat sciatic nerve^6^.

Gold nanorods (AuNRs) are extremely unique in having an optically active surface, caused by localized surface plasmon resonance^7^. It has been reported that activation of this feature of AuNRs leads to enhanced axonal extension of the NG108-15 neuronal cells^8^; axonal enhancement is connected with a moderate increase in intracellular calcium (Ca^2+^) concentration^9^. It is believed that the excitation of the active plasmonic surface could produce transient heating, which in turn would change the membrane capacitance and activate specific sensitive ion channels located in the cell membrane^10^.

Several studies have explored the role of AuNRs in enhancing cellular activity^8–14^. For example, Paviolo *et al*.^8^ observed a noticeable increase in neurite length when NG108-15 neuronal cells were posed to cell culture medium containing 5% (v/v) AuNRs solution. The study compared differences when cells were exposed to pure AuNR versus poly (4-styrenesulfonic acid) - and silica-coated AuNRs. The incubated cells were exposed to 1.2–7.5 W/cm^2^ laser at 780 nm to stimulate neuronal cells. In another study, Yong *et al*.^11^ claimed that the laser-induced heating of AuNRs can stimulate electrical activity in auditory neurons. They demonstrated this by incubating rat primary auditory neurons with a medium culture containing silica-coated AuNRs and silica-coated gold nanospheres. The incubated cells were exposed to a near-infrared laser at 780 nm to stimulate the primary neurons.

Even though these procedures demonstrate the potential of AuNRs in generating a neural response, the methods are not adequate for preparing a 2D substrate and ultimately a 3D scaffold. Furthermore, it is technologically challenging to incorporate AuNR solution that has been added to the cells in previous studies and hence, cannot be used to form a physical device to treat nerve injuries. Additionally, the studies described above, do not provide real data about the interaction between the cell membrane and the substrate, since this interaction is based on cellular encapsulation of the AuNRs rather than cellular adherence.

It has been demonstrated in recent years that adult-tissue derived mesenchymal stem cells (MSCs) are valuable for cell-based therapies in tissue engineering and regeneration. This is primarily due to their inherent multipotent potential to undergo differentiation into osteocytes, adipoctyes, chondrocytes, myocytes, and hepatocytes. Human MSCs (hMSCs) can be isolated from any adult tissue, including, bone marrow, peripheral blood, dental pulp, synovial fluid, synovial membrane and adipose tissue. Human adipose-tissue is a relatively easy, and noninvasive source of MSCs, and is the preferred source in human medicine^15–18^. In the last decade or so, it has been established that hMSCs also have the potential to differentiate into electrically functional neural cells, which can easily translate into novel strategies for the treatment of neurological disorders and/or peripheral nerve injuries^19–21^ Since, the adhesion, proliferation, and differentiation potentials of MSCs can be significantly affected by biomaterials^22,23^, it is imperative that these properties are tested before the cell/biomaterial constructs are implanted in an animal. Using *in vitro* cell proliferation and differentiation assays, these properties can be tested reliably prior to clinical applications.

Although there are several reports suggesting the potential to use AuNRs in regenerative medicine, all studies thus far, are focused on the *in vitro* based analyses of immortalized cell lines, like NG108-15 neuronal cell^8^, or Schwann cells^14^. Similar assays assessing the differentiation potentials of hMSCs in presence of neuromimetic AuNRs are lacking. It has been suggested that the physical and chemical properties of biomaterials can significantly affect the adhesion, proliferation and differentiation abilities of hMSCs, and hence, it is imperative that these properties are tested *in vitro* prior to their application *in vivo*.

In this study, we established a novel 2D nanocomposite system that incorporates AuNRs as an active layer for cell adhesion, proliferation, and cell-substrate interaction. Based on previous reports, AuNRs with an aspect ratio 3 were chosen as the building blocks to form a 2D substrate. A novel and relatively simple procedure was used to produce a 2D system of where cell-substrate interactions could be easily investigated. By utilizing polyethylene glycol (PEG), the surface chemistry was controlled in such a way that a coating of AuNRs was successfully deposited on the substrate. By using a negatively charged substrate and positively charged AuNRs, strong adhesion was achieved. Moreover we expect that the positive nature of the free terminal amine (opposite the substrate) on the AuNRs will enhance the cellular adhesion and thus, their interactions. The ability to modify surface chemistry of AuNRs, by changing the terminal groups of PEG, opens a new route to combine a layer of AuNRs with immortalized and primary cultures of cells, such as progenitor cells, mesenchymal stem cells and Schwann cells, which could lead to new cellular therapeutic neural devices.

In the present report, the 2D system of AuNRs was prepared and used to study the effect of neural differentiation of hMSCs. The cytocompatibility of the AuNRs was first tested using RT4-D6P2T, a commercially available immortalized Schwann cell line. Subsequently, the neural differentiation of hMSCs was evaluated *in vitro*, by assessing the expression patterns of Vimentin, glial fibrillary acidic protein (GFAP) and a calcium binding protein B (S100β), specific to the mesenchymal and the neural progenitor cells. Since, one of the first steps in neural development includes differentiation of neurons from neural stem cells or progenitor cells, this study is the first step before the AuNRs can be implemented *in vivo*.

## Methods

### Materials

All biochemicals, cell culture supplements, and disposable tissue culture supplies were purchased from Thermo Fisher Scientific unless otherwise noted. In all preparation steps, deionized (DI) water from a Siemens Labostar unit with a resistance of 18 M/cm was used. Sodium borohydride (99%), gold (III) chloride trihydrate (99%), L-ascorbic acid (98%), *N*-(3-Dimethylaminopropyl)-*N*′-ethylcarbodiimide hydrochloride (EDC), and N-hydroxysuccinimide (NHS) were purchased from Sigma-Aldrich. Cetyltrimethylammonium bromide (CTAB 99%) was purchased from MP Biomedicals. HS-PEG and HS-PEG-NH_2_ were purchased from Nanocs Inc.

### Preparation of gold nanorod (AuNR) substrate

AuNRs with dimensions of ~12 nm diameter and ~36 nm length were prepared according to the seed-mediated method described earlier^24^.

**Figure 1:**
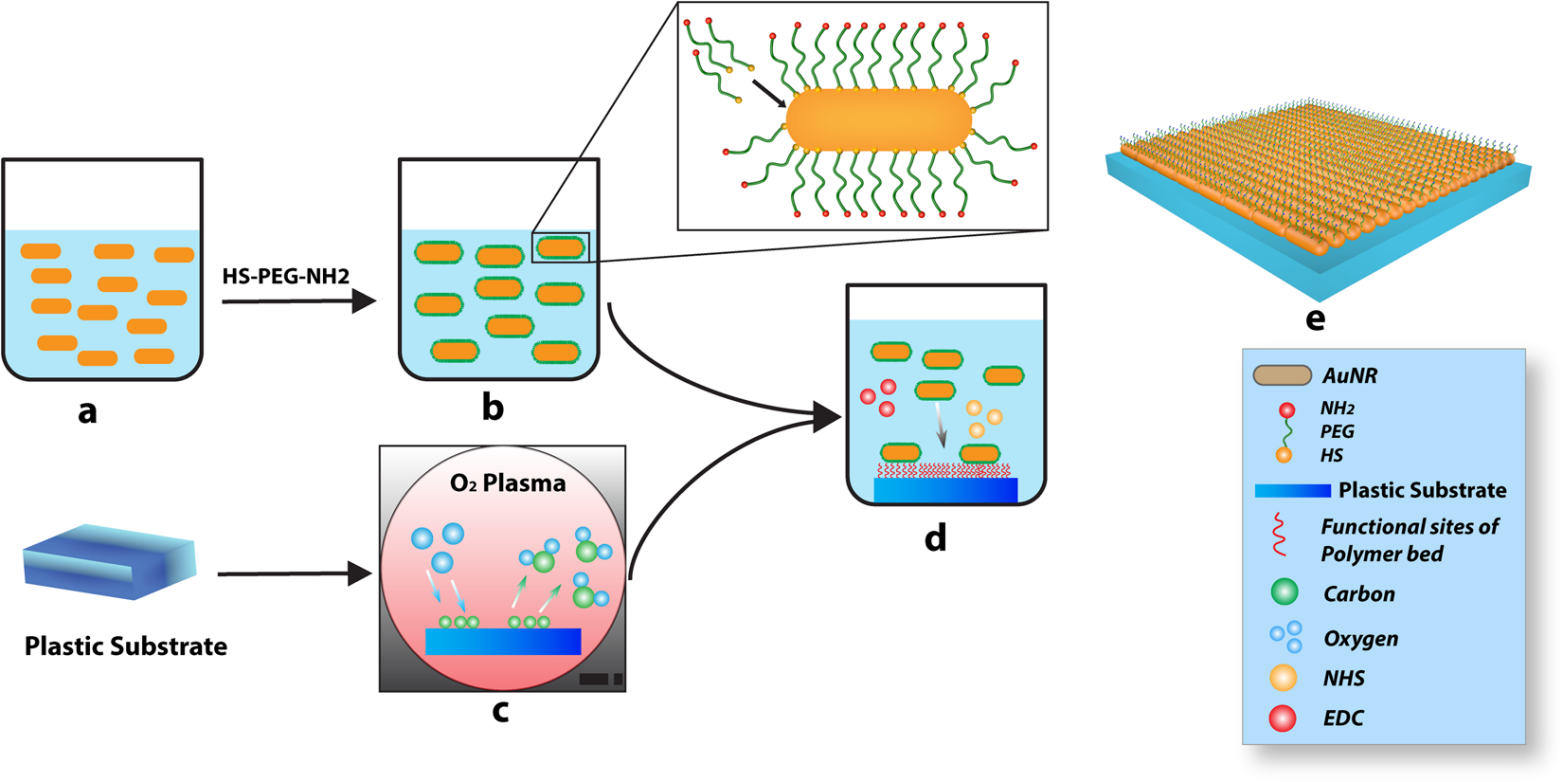
General procedure used to prepare AuNRs 2D nanocomposite. (**a**) Preparation of AuNRs, (**b**) Functionalization of AuNRs, (**c**) O2 plasma treatment to the plastic Thermanox substrate, (**d**) Assembling the functionalized AuNRs over the plastic Thermanox substrate, (**e**) Washing with Di water, and then treatment with ethanol and UV for sterilization.

The purified AuNRs (Fig. 1a) were then functionalized with HS–PEG–NH_2_ (Fig. 1b) to modify their surface chemistry and has been previously reported^7,25^.

Commercially available 15 mm diameter Thermanox coverslips were used as the substrate to coat functionalized AuNRs. Prior to coating, the substrate was treated with oxygen plasma (100 mTorr O_2_ with 65 W) for 30 minutes (Fig. 1c). This step produced a more hydrophilic surface and induced reactive oxygen functional groups^26^. Subsequent to the plasma treatment, NHS/EDC conjugation assay^27^ was performed to cover the substrate with a coating of AuNRs-HS-PEG-NH_2_ (Fig. 1d). Briefly, 10 mg/mL stock solutions of EDC and NHS each were prepared in 1xPBS. 2 ml of 2 mg/ml functionalized AuNRs were added to each of the O_2_-treated Thermanox cover slips, followed by the addition of 228 μl of EDC solution and 112 μl of NHS solution. Using an orbital shaker, the mixture was gently shaken at 100 rpm for 4 hours in order to achieve full conjugation. The excess liquid above the substrate was removed, and the substrate was washed extensively with DI water to remove any unbound AuNRs-SH-PEG-NH_2_ molecules. A virtual design depicting the final product is shown in Fig. 1e.

### Characterization of AuNRs

The size, structure and morphology of AuNRs were observed using a JEOL-2100F transmission electron microscope with an accelerating voltage of 80 kV, and a JEOL JSM7000F scanning electron microscope. For TEM, a few drops of AuNRs and AuNR-SH-PEG-NH_2_ suspended in ethanol, were deposited on carbon coated copper grids, and dried at room temperature for 30 minutes. For SEM, substrates were coated with a thin carbon layer for visualization.

UV-Vis-NIR spectra was recorded on a UV 3600 Shimadzu spectrophotometer to determine the transverse and longitudinal peaks for AuNRs, both in solution and in the substrate form. A 500 µg/ml solution of AuNRs was used to procure the measurement in solution, while the thin film measurement method was used for the substrate. All measurements were taken between 400–1000 nm.

Electrophoretic mobility (zeta-potential) of the nanoparticles was determined using a Zeta Reader (MARK 21). Briefly, in deionized water, 500 µg/ml of pure and functionalized AuNRs were loaded in 5 ml syringes, and the data was collected at an applied potential of 20 eV.

X-ray photoelectron spectroscopy analysis was conducted with a Thermo Scientific Model K-Alpha XPS instrument using monochromatic Al K_α_ radiation (1486.7 eV) with an output power of 36 W. Data were collected and processed using the Thermo Scientific Avantage XPS software package. Spectra were charge-corrected using the main C1s peak due to adventitious hydrocarbon set to 284.80 eV. Peak fitting was performed using mixed Gaussian/Lorentzian peak shapes and a Shirley/Smart type background.

### Immortalized Schwann cells

An immortalized Schwann cell line, RT4-D6P2T was obtained from the American Type Culture Collection. A second passage of RT4-D6P2T cells, were incubated for 7 days over the AuNRs substrates, and used in the experiments described below. Tissue culture treated Thermanox cover slips were used as positive controls. Cells were incubated at 37 °C (5% CO2, 95% air) in DMEM medium supplemented with 10% fetal bovine serum (ATCC, 63310972) and 1% penicillin/ streptomycin. The medium was changed every two days.

RT4-D6P2T cells seeded on the AuNRs were evaluated for their viability, adherence, morphology and the expression of Schwann cell protein markers using the WST-1 and immunofluorescence assays, respectively.

### WST-1 assay

WST-1 Cell Proliferation Assay Kit was used to determine the mitochondrial metabolic activity of RT4-D6P2T cells, and thus, was used as a measure of cell viability as per the manufacturer’s instructions. The reconstituted WST-1 mixture was added to each well in a 1:10 dilution, and incubated for 2 hours in the dark at 37 °C. A microplate reader was used to measure the absorbance at 450 nm. Wells containing the AuNRs substrate, media, and WST-1 reagent alone were measured as a background controls. All assays were performed in triplicate and data was analyzed using Students’ TTest analysis. P value of <0.05 was considered statistically significant.

### Immunofluorescence

For immunofluorescence, all cells at specified time points were fixed with 4% paraformaldehyde at room temperature for 10 minutes; rinsed twice with HBSS (Hanks’ Balanced Salt Solution), and then permeabilized with 0.1% Triton X-100. Cells then blocked with a Universal Blocking Reagent for 30 minutes at room temperature. Cells were subsequently incubated with 2µgs of primary antibodies for S100β, GFAP and Vimentin (BD Pharmingen) at 4 °C for 24 hours. Subsequently, cells were washed twice with HBSS and incubated with appropriate Alexa Fluor secondary antibodies at room temperature for 30 minutes in dark. The cells were then mounted with Prolong Gold antifade reagent with 4′, 6-diamidino-2-phenylindole (DAPI). The cells were imaged and captured with a laser scanning spectral confocal microscope (Leica TCS SP2) at 20x magnification.

### Isolation, and *ex vivo* expansion of human MSCs

Human adipose tissue was obtained from patients undergoing panniculectomies in accordance to a protocol approved by the IRB at the University of Tennessee Medical Center. Informed client consent was obtained prior to the harvest. After resection, the adipose tissue was immediately processed as previously described^28^ The cells were grown to 80–90% confluence and then harvested with 0.05% trypsin/EDTA, for cryopreservation (80% FBS, 10% DMEM/F12, 10% DMSO) or split and seeded into new flasks for expansion. All experiments were performed using cells from passage 2–6 in complete growth media (CGM) (DMEM/F12, 1% penicillin-streptomycin/amphotericin B, 10% FBS).

### Characterization of human MSCs

Isolated human MSCs were characterized by a combination of specific cluster-of-differentiation (CD) markers expressed on their cell surface. Approximately 1 × 10^6^ human MSCs were used to stain cells with: anti-human CD29-PE/CD44-APC, CD73-PE/Cy7, CD90-Alexa-fluor 647/CD105-PE, CD34-Alexa-fluor 647/CD45-PE, CD106-PE/HLA-DR-APC or their corresponding isotype matched controls (Biolegend). Antibodies were used at the manufacturer’s recommended concentrations. Cells were harvested, counted, blocked in 1% goat serum in PBS for 20 minutes at room temperature then stained with each antibody for 20 minutes at room temperature in darkness. Cells were washed with PBS, collected by centrifugation and fixed with 4% paraformaldehyde/PBS for 10 minutes at room temperature in darkness. Cells were resuspended in PBS and 20,000 events were measured using a BD FACS Calibur. The raw data was analyzed by FlowJo software.

Human MSCs were also evaluated for their ability to undergo tri-lineage differentiation, *in vitro*, using the following differentiation cocktails in CGM: Osteogenic (100 nM dexamethasone, 10 mM β-glycerol phosphate and 155μM ascorbic acid), Adipogenic (1μM dexamethasone, 500μM 3-isobutyl-1-methylxanthine (IBMX), 5μg/ml recombinant human insulin, 621μM ascorbate-2-phosphate and 60μM indomethacin), Chondrogenic (100 nM dexamethasone, 155 nM ascorbate-2-phosphate, 20ng/ml TGF-β1, 1X ITS-G, 50μg/ml proline and 1 mM sodium pyruvate). In all differentiation experiments, non-induced (i.e. cells grown in the absence of any differentiation media) were used as controls. At 21 days, cells were fixed in 4% paraformaldehyde/PBS for 10 minutes at room temperature. Alizarin red, oil-red-o and alcian blue were used to detect calcium, lipid and glycosaminoglycan deposition, respectively, in cells undergoing osteogenesis, adipogenesis and chondrogenesis, respectively. All images were obtained using a Zeiss Axiovert 40 C microscope (Carl Zeiss) equipped with Nikon Digital Sight DS-Qi1Mc camera (Nikon Instruments).

### Differentiation of human MSCs into neural-like cells

Human MSCs isolated above were induced to undergo differentiation into neural-like cells as described earlier^29^. Neural differentiation was induced in CGM supplemented with 0.5 mM 3-isobutyl-1-methylxanthine (IBMX)/1 mM dibutyryl cAMP (dbcAMP) for 24 h, and 6 days. Media was replenished every 3 days, and differentiation was evaluated by the changes in cell morphology during the induction process.

The expression of GFAP and S100β was evaluated in the undifferentiated and neural-like differentiated hMSCs in presence of AuNRs using immunofluorescence. Imaging of Vimentin was used to assess morphological changes in mesenchymal cells morphology during differentiation. All cells were fixed at 24hrs and 6 days post differentiation. AuNRs alone without any cells served as negative controls, whereas, cells seeded on the glass coverslips alone were included as the positive controls.

## Results and Discussion

### Preparation and characterization of AuNRs

A relatively simple layer technique was used in the preparation of AuNRs 2D nanocomposite system. Gold nanorods were functionalized with PEG and containing free amine groups were layered on a plastic plasma treated surface. The nanocomposite thus generated was used in cell adhesion and neural differentiation assays.

Figure 1 illustrates the strategy used in the preparation of AuNRs. A plastic substrate (Thermanox) was treated with oxygen plasma to increase the hydrophilicity of the substrate, and to “activate” the surface with oxygen groups (such as those present in carboxylic acid) that can form covalent bonds with the surface groups of the AuNRs. There are two forces which might hold the functionalized AuNRs with the polymer substrate: (1) hydrophilic interactions between PEG polymeric chains and the hydrophilic polymer substrate and (2) covalent bonds between functionalized AuNRs and the substrate surface. The AuNRs (with aspect ratio of 3, average diameter of 12 nm & average length 36 nm) are functionalized with PEG that have terminal amine functional groups which form amide bonds with the plastic substrate through EDC/NHS reaction.

**Figure 2:**
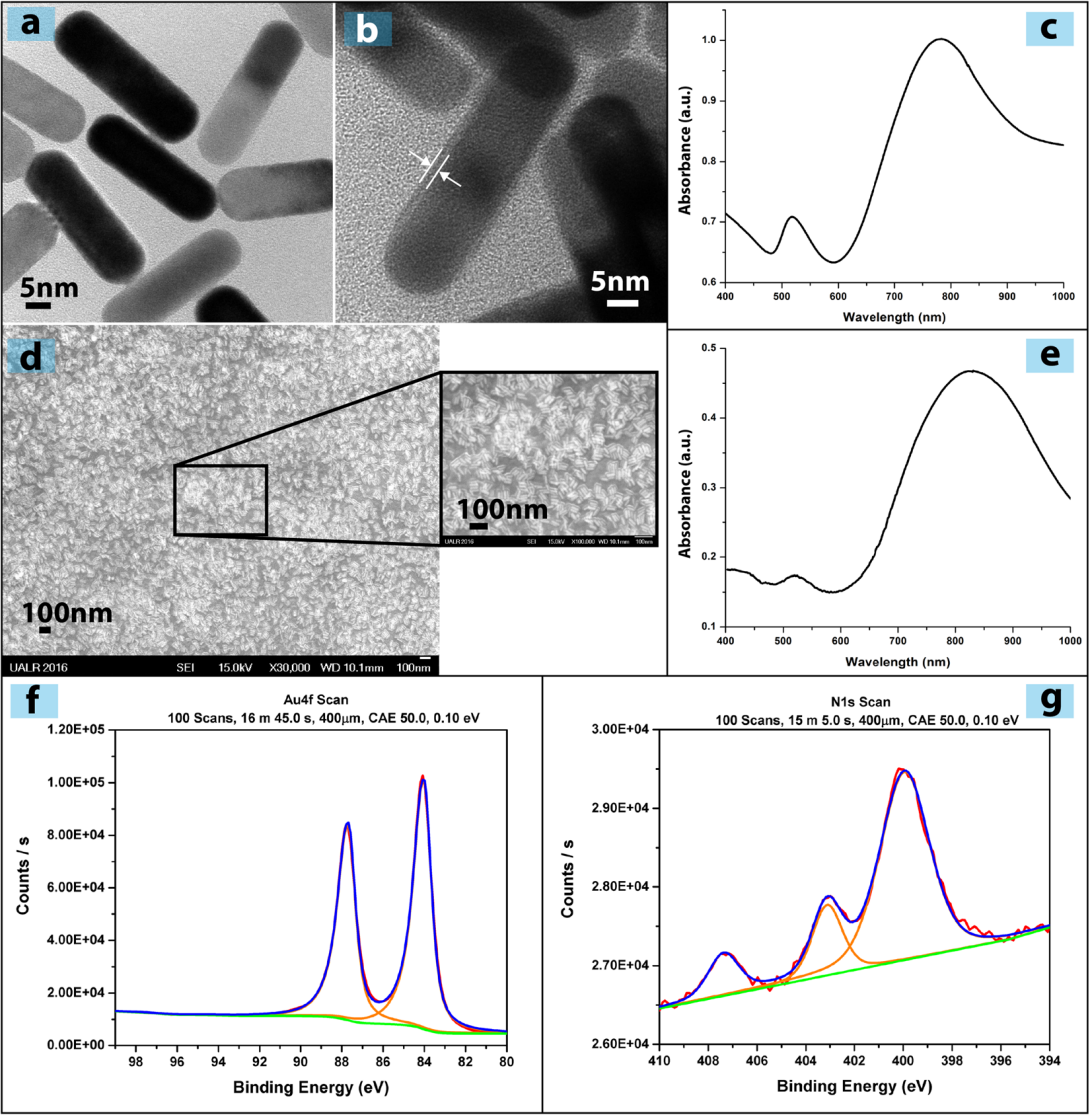
Characterization of AuNRs 2D nanocomposite. (**a**) TEM images of AuNRs; (**b**) TEM images of functionalized AuNRs. The white arrows refer to the PEG shield; (**c**) UV-Vis spectrum of functionalized AuNRs; (**d**) SEM images of the Thermanox coated with AuNR-NH_2_; (**e**) UV-Vis spectrum of AuNRs on the Thermanox; (**f**) The XPS spectra of Au4f; (**g**) The XPS spectra of N1s.

AuNRs were characterized using a combination of different physicochemical methods. As shown in Fig. 2a, native AuNRs with ~12 nm diameter, ~36 nm length, were confirmed by electron microscopy. In order to control the fabrication of AuNRs layer on the plastic substrate, the surface chemistry of AuNRs should be modified. Generally, the common way to modify the Au nanoparticles is to attach thiol molecules over Au surface by strong semi-covalent Au–S bonds^7^. TEM and zeta potential were used to investigate the presence of Thiol-PEG-Amine (SH-PEG-NH_2_) layer on the surface of the AuNRs. After functionalization approximately a 1 nm shield can be observed on the AuNR in Fig. 2b thus, confirming the assembly of AuNR with the HS–PEG–NH_2_ group. As a result of water absorbed by the PEG chains, the effective thickness of the AuNR particle is expected to be larger than 1 nm. Although the TEM indicated a 1 nm shield of SH-PEG-NH_2_ of the AuNRs, this is observed only under vacuum conditions since, the PEG chains collapse under dry or semidry conditions^30^. On the other hand, PEG chains tend to expand in non-dry condition as a result of water absorbed. The UV-Vis spectrum (Fig. 2c) of the functionalized AuNRs shows the expected surface plasmon profile (λ_max_ at 520 nm and 780 nm) as described in previous published work^31^. Moreover, the zeta-potential of pure AuNRs was −15mV and after functionalization with HS–PEG–NH_2_ it shifted to +24 mV, confirming an exterior positive surface change due to terminal amine groups.

The assembly and attachment of AuNR-SH-PEG-NH_2_ to the plastic Thermanox substrate and the presence of the NH_2_ terminal over the AuNR layer was confirmed using SEM, XPS, and UV-Vis spectroscopy. The SEM images in Fig. 2d show a coating of AuNR-SH-PEG-NH_2_ over the plastic substrate even though the AuNR-SH-PEG-NH_2_ nanorods do not align uniaxially as proposed in the virtual design Fig. 1e. The UV-Vis spectrum for the layer of AuNRs-SH-PEG-NH_2_ on the plastic substrate shown in Fig. 2e, indicates that this structure has both transverse and longitudinal plasmon resonance peaks, located at 520 and 830 nm, respectively. Since the longitudinal peak is more prominent than when the functionalized AuNRs are in solution it is evident that the AuNRs are laying on their long axis. Furthermore, the longitudinal surface plasmon is significantly shifted toward the IR region compare to control (the AuNRs dispersed in DI water, where UV-Vis shows transverse and longitudinal plasmon resonance peaks located at 520 and 790 nm). The shift in longitudinal plasmon resonance peak is attributed to aggregation of AuNRs on the substrate; this change in plasmonic resonance peak has been explained previously in detail^32^.

The XPS spectra of Au4f and N1s from the constructed layer of AuNR-SH-PEG-NH_2_ are given in Fig. 2f and g. The sampling depth of our XPS system for metals, which is defined as three times the mean free path of electrons, is approximately 3–3.5 nm. The organic SH-PEG-NH_2_ shell is ~1 nm thick, as shown by TEM. The Au4f spectra clearly demonstrates the existence of Au atoms in the nanorods^33^. The collected spectra also indicates an Au4f5/2 binding energy of 83.42 eV, as shown in Fig. 2f. The N1s binding energy value of ~400 eV attributed to NH_2_ bonds^34^ is used as an evidence of a functionalized organic layer as shown in Fig. 2g.

The results presented here reflect the successful preparation of the AuNRs 2D nanocomposite system and the attachment of functionalized AuNRs to the plastic substrate. There are many advantages of combining the AuNRs with SH-PEG-NH_2_. The AuNR-SH-PEG-NH_2_ system can be easily used to form coatings over various plastic surfaces, either by interaction between the NH_2_ terminal of SH-PEG-NH_2_ with functional sites of the O_2_ plasma treated plastic substrate, or by hydrophilic interactions between the hydrophilic polymer substrate with AuNR-SH-PEG-NH_2_. Surface hydrophilicity, which has been achieved by the incorporation of PEG^35^ within this configuration, is an important feature or enhanced cell interaction^36,37^. The free terminal amine of the functionalized AuNRs also provides a positively charged surface, an environment favorable for cell interaction, and improved neuronal growth^36^.

### The AuNRs are cytocompatible with RT4-D6P2T cells

**Figure 3:**
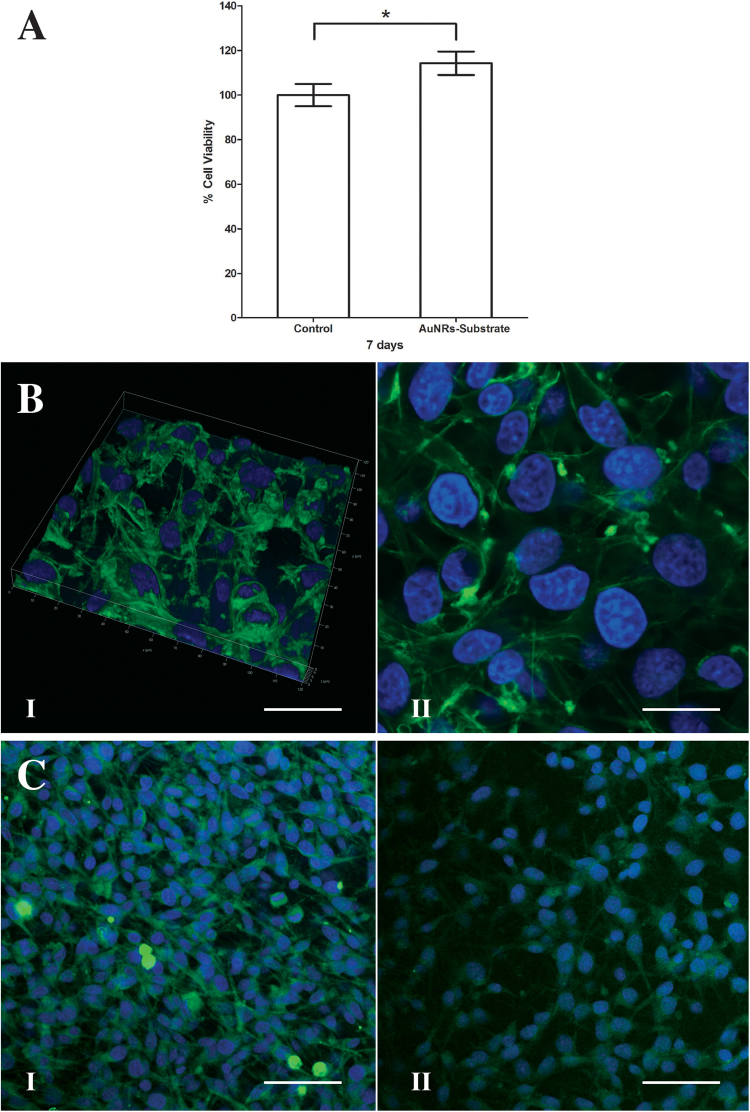
Schwann cells on AuNRs 2D substrate. (**A**) WST-1 assay. The viability of RT4-D6P2T cells was assessed on AuNRs 2D nanocomposite after incubating for 7 days. A 14% statistically significant increase in cell viability on AuNRs relative to Thermanox tissue culture treated cover slips was observed. Error bars represent SD. Asterisk indicates P < 0.05. (**B**) Cell adhesion. Representative confocal images show the actin cytoskeleton of RT4-D6P2T cells stained with Alexa Fluor 488 Phalloidin after incubation on AuNRs for 7days. (I) 3D Z stacking shows cell adhesion and spreading, (II) a representative confocal image to show the expression of actin (green) in RT4-D6P2T cells. DAPI was used to stain the nucleus (blue). (**C**) Expression of marker proteins. Representative confocal images show the expression of S100 (I) and GFAP (II) when RT4-D6P2T cells adhere and proliferate on AuNRs substrate for 7 days. S100 β and GFAP (green) and DAPI (blue) was used to stain the nucleus.

The cytotoxicity of the AuNRs was assessed by evaluating cell viability and the fluorescent staining of the cell cytoskeleton using WST-1 assay (Fig. 3A) and phalloidin Alexa fluor 488 staining of F-actin (Fig. 3B), respectively. The RT4-D6P2T cells were seeded on the AuNRs and assayed after 7 days. As shown in Fig. 3A, there was a 14% significant increase in cell viability on AuNRs compared to the Thermanox tissue culture treated surface (P = 0.005451393). Immunofluorescence results showed a high actin cytoskeleton organization in the cells at the same time point. Z stacking showed that the cells adhered and spread uniformly onto the surface of AuNRs while still maintaining their structural integrity. The WST1 assay and the actin staining results confirm that AuNRs are biocompatible, and do not release any molecules toxic to the cells within the one week study period and thus, can be used as a substrate to support cell adhesion.

In addition to the above, immunofluorescence was also used to evaluate that the Schwann cells maintained the expression of their protein markers, S100β and GFAP, thus, maintaining their integrity on AuNRs (Fig. 3C).

### Characterization of hMSCs

**Figure 4:**
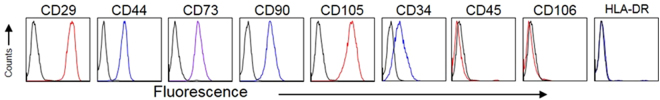
Immunophenotyping of hMSCs by flow cytometry. Human MSCs were stained with the indicated antibodies and then analyzed by flow cytometry. Cells strongly express the markers (CD29, CD44, CD73, CD90, CD105) associated with the mesenchymal stem cells, while expression of hematopoietic (CD34, CD45, HLA-DR) and endothelial (CD106) markers is comparatively less at passage 2 and markedly reduced at passage 6. CD34 and CD45 are hematopoietic cell markers whereas, the HLA-DR is the major histocompatibility complex II protein, primarily involved in T cell activation and proliferation. Black open histograms indicate isotype matched controls for each antibody; colored open histograms represent positive reactivity with the indicated antibodies.

In order to maintain uniformity in the classification of progenitor cells, cells are classified as MSCs if they satisfy certain criteria^15^ One is to adhere and proliferate on polystyrene treated surface with a spindle-like fibroblastic morphology, and second is to express a combination of specific cluster-of-differentiation (CD) markers. Subjective evaluation demonstrated fibroblastic morphology of cells during *in vitro* culturing and serial passaging from passage 1 through 6. Flow cytometric analyses revealed that >99% cells isolated and expanded in culture from the human adipose tissue were positive for CD29, CD44, CD73, CD90 and CD105 (Fig. 4). The endothelial surface marker, CD106, showed minimal antigenic reactivity (4.68%) in passage 2 cells, while the hematopoietic marker, CD34, showed 61.4% expression. In passage 6 cells, the expression of CD34 and CD106 was reduced to 11% and 1.75% respectively. Similarly, the passage 2 cells were <3% positive for the monocyte-macrophage marker, CD45, and the human MHC Class II marker, HLA-DR, the levels of which were nearly undetectable in passage 6.

**Figure 5:**
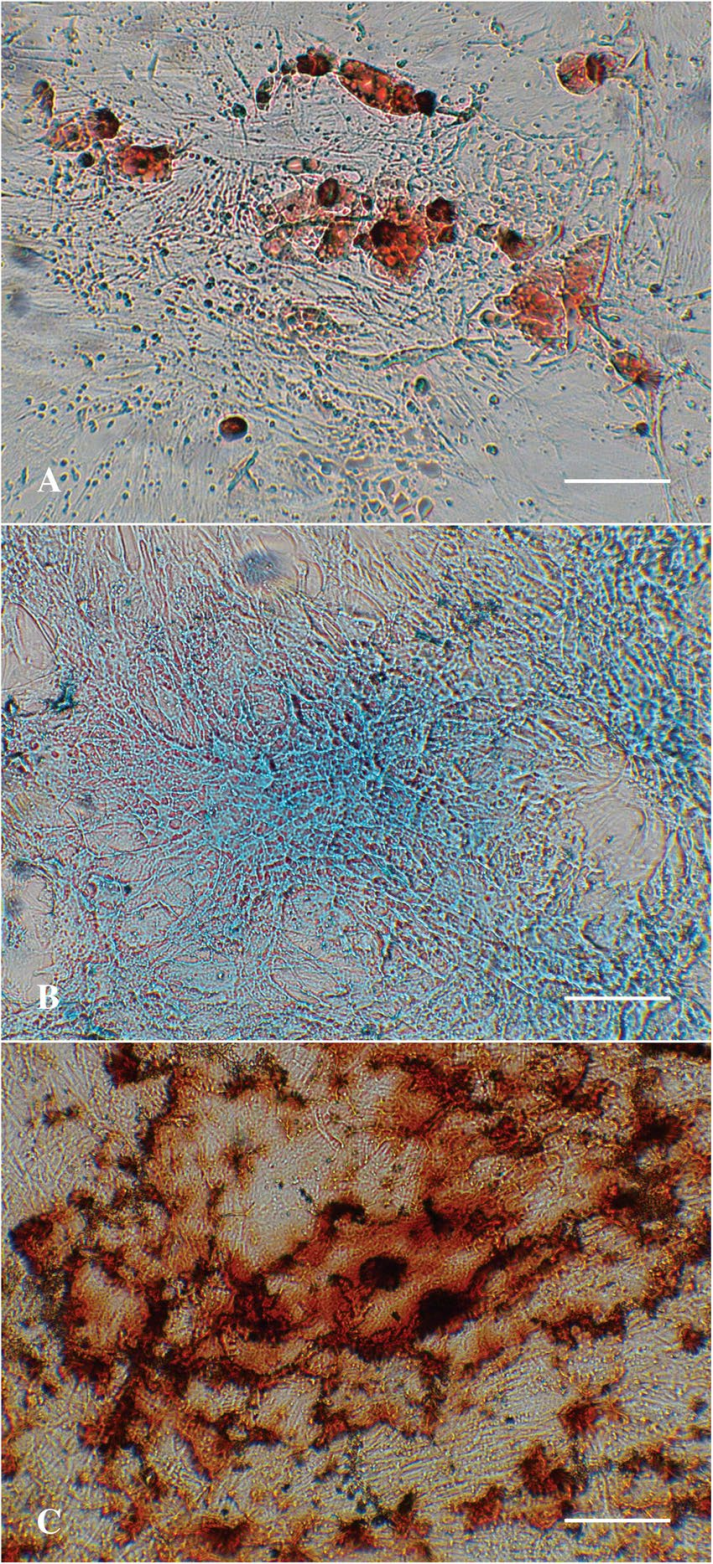
Tri-lineage differentiation assays of hMSCs. Representative images of the oil-red-o, alcian blue and alizarin red staining demonstrating adipogenesis, chondrogenesis and osteogenesis, respectively, after *in vitro* differentiation.

The third criteria to identify cells as MSCs is to demonstrate their potential to undergo tri-lineage differentiation *in vitro*. As shown in Fig. 5, when the isolated MSCs were incubated in a lineage-specific inducing cocktail they did undergo differentiation into the expected cell types compared to the uninduced control cells. Alizarin red, oil-red-o and alcian blue staining confirmed the presence of calcium, lipids and glycosaminoglycans, respectively, when the MSCs revealed osteogenesis, adipogenesis and chondrogenesis, respectively. Thus the isolated MSCs met the criteria for having the multipotential to undergo tri-lineage differentiation.

**Figure 6:**
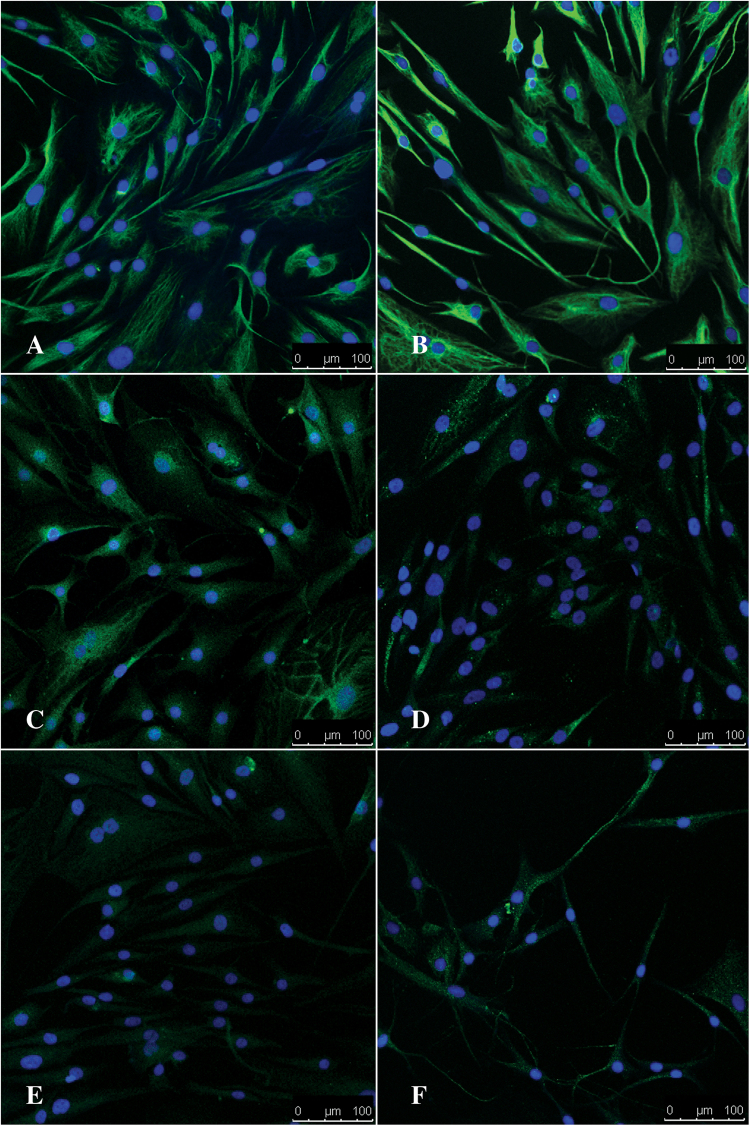
Differentiation of hMSCs into cells of neural lineage on tissue culture surfaces. Representative confocal images show the expression of Vimentin, S100β and GFAP at 24 hours (**A**,**C**,**E**) and 6 days (**B**,**D**,**F**) post differentiation. Scale bar = 100 µm. Note the bipolar morphology of the cells in A and B.

In order to evaluate the potential of hMSCs to undergo differentiation into cells of neural lineage and subsequently to assess their behavior on AuNRs, hMSCs were first differentiated into a neural cell-like phenotype on a polystyrene-coated tissue culture dish, and the expressions of Vimentin, S100β, and GFAP were confirmed using immunofluorescence after 24 hrs and 6 days of differentiation. The *in vitro* patterns of expression were as expected (Fig. 6). Vimentin was expressed at 24h and 6days post differentiation (Fig. 6A,B) indicating the maintenance of cell shape, integrity of the cytoplasm, and stable cytoskeletal interactions in hMSCs during differentiation. Noteworthy is the bipolar morphology of the cells indicating that the hMSCs were becoming neural-like as early as 24 hours in presence of cAMP and IBMX. This is further confirmed by the relatively higher expression of S100β in the 24 hr sample (Fig. 6C), and of GFAP after 6 days (Fig. 6F).

**Figure 7:**
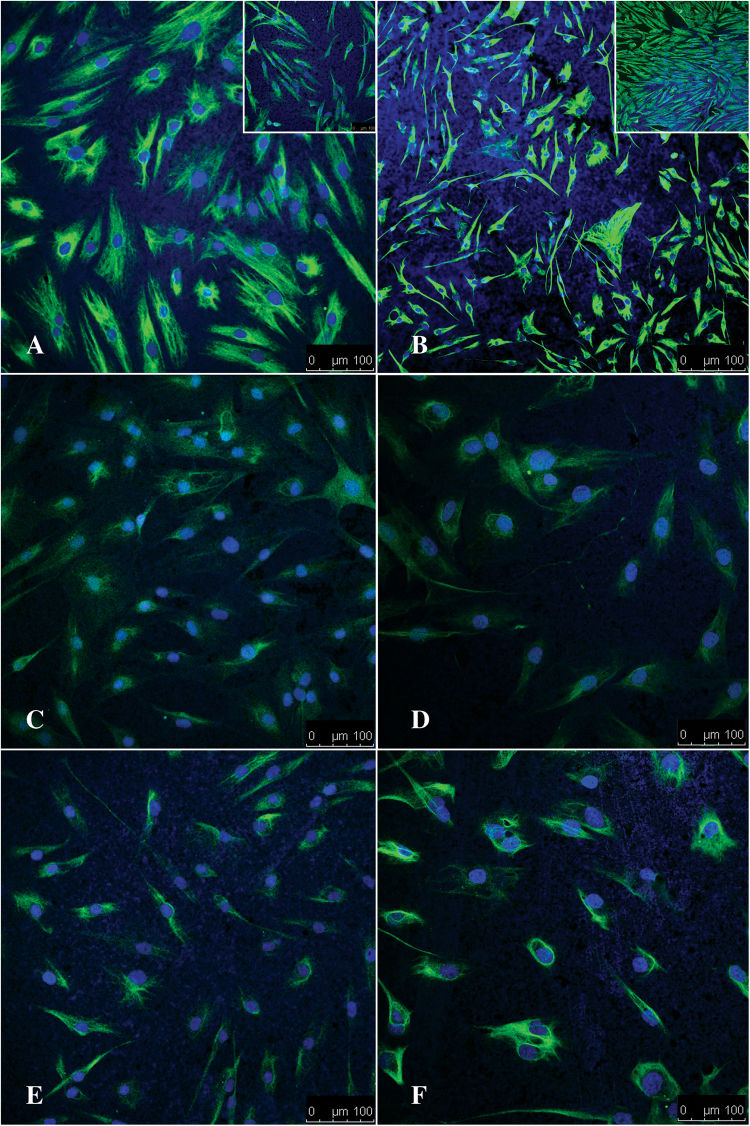
Differentiation of hMSCs into cells of neural lineage on AuNRs 2D nanocomposite. Representative confocal images show the expression of Vimentin, S100β and GFAP at 24 hours (**A**,**C**,**E**) and 6 days (**B**,**D**,**F**) post differentiation on AuNR substrate. The insets in A and B show the corresponding undifferentiated controls at 24 hours and 6 days. Scale bar = 100 µm.

The hMSCs were next seeded on the AuNRs and differentiated into neural lineage under conditions described above, and analyzed after 24 hrs and 6 days for biomarkers as described above (Fig. 7). As expected, Vimentin was expressed at 24hrs and 6 days displaying the bipolar morphology, integrity of the cytoskeleton and the cytoplasm of cells as early as 24 hrs. and maintaining it until 6 days (Fig. 7A,B) in the differentiation medium. Importantly, an increase in cell density as visualized in the undifferentiated control cells (Fig. 7A,B insets), further confirmed and supported the WST-1 assay data, that the AuNRs are not cytotoxic to the hMSCs. The expression patterns of S100β and GFAP on AuNRs were interesting. Unlike, the expression pattern on the polystyrene surfaces, the S100β was expressed after 24hrs of differentiation and its expression was maintained even after 6 days (Fig. 7C,D). GFAP was expressed in hMSCs on AuNRs as early as 24hrs and persisted even after 6 days, suggesting that the differentiation process was accelerated in presence of AuNRs.

Neurogenesis is the process by which neurons are generated from neural stem cells and progenitor cells. It plays a central role in neural development. Neurogenesis is a complex process that is controlled by a variety of key proteins and their targets^38^. The S100 protein family is the largest subgroup of the calcium –binding EF hand protein group^39^. S100β, is one member of this family, considered to be an important neuro-biomarker and which regulates the cytoskeletal structure, cell proliferation and neural differentiation. These actions of S100β may be modulated via the MAPK or the NF-kβ signaling pathways. GFAP, a class-III intermediate filament, is a cell-specific marker, solely expressed in astrocytes, and used to distinguish astrocytes from other glial cells during development^40^. In summary, the results presented in this study, demonstrate that the early expression of GFAP when hMSCs differentiate on AuNRs in presence of the inducers like cAMP and IBMX, indicates that their differentiation was accelerated. The morphological changes in MSCs coincided with the increase in expression of both GFAP and S100β. The cells developed neural-like characteristic morphologic features in presence of AuNRs within 24 hrs of seeding, and the expression of GFAP was detected as early as 24hrs indicated that the naïve, undifferentiated hMSCs were differentiating into astrocytes as early as 24hrs rather than 6 days as observed on polystyrene tissue culture surfaces.

Researchers have demonstrated that cells exhibit many of the characteristics of neuroendocrine cells *in vitro* when the intracellular cAMP levels are elevated by adding inducers like, epinephrine, isoproterenol, forskolin, and IBMX to the culture medium^41–43^. Specifically, neuron-like cells differentiated from MSCs exhibited increased cytoplasmic Ca^2+^ levels^44^. Hence, it is reasonable to speculate that the surface properties of the AuNRs in conjunction with the chemical cues that are triggered in the hMSCs, may cause the cells to progress towards the neural lineage. The signaling mechanisms resulting in these changes are unknown. Future investigations are required to study the mechanism(s) of these cellular changes in the AuNR + hMSC nanocomposite system.

## Conclusion

Figuress study, a novel protocol was developed to form a layer of 2D AuNRs-SH-PEG-NH_2_ nanocomposite on a plastic surface (Fig. 1). Such a structure will allow us to investigate the role AuNRs as a bioactive substrate, by evaluating cell-substrate interaction. The AuNRs were of 12 nm diameter, functionalized with thiolated PEG and contained –NH_2_ groups for enhanced cell adhesion (Fig. 2). Immortalized, commercially available Schwann cells adhered to and were viable on AuNRs and maintained their cellular integrity for 7 days (Fig. 3), confirming the lack of cytotoxicity of the materials. Finally, the AuNRs were evaluated as a suitable substrate for neural differentiation of human MSCs. Human MSCs were isolated from the human adipose tissue and were characterized using standard cellular assays (Figs 4 and 5). Finally, the differentiation of hMSCs into neural cells was compared in presence and absence of AuNRs using the expression of three target proteins, Vimentin, S100β and GFAP (Figs 6 and 7). The expression pattern of Vimentin confirmed that the cells maintained their fibroblastic morphology, and depicted changes when neural differentiation was induced. The expression patterns of S100β and GFAP suggested that the process of neural differentiation was accelerated in presence of AuNRs. This observation is important and necessary for the development and design of the 3D scaffold of AuNRs.

A major finding of this study is that the surface plasmon of the AuNRs nanocomposite system can be activated by an IR laser source. This activated surface can thus, potentially promote cell growth and neural differentiation by opening Ca + 2 ion channels, thereby, modulating the neurogenesis process. Hence, future experiments to use the 3D AuNRs scaffold with improved potential for neural differentiation in an *in vivo* animal model and to evaluate the signaling mechanism(s) of neural differentiation of human MSCs in presence of AuNRs, should be carried out.
